# Spatial Genomic Resource Reveals Molecular Insights into Key Bioactive-Metabolite Biosynthesis in Endangered *Angelica glauca* Edgew

**DOI:** 10.3390/ijms231911064

**Published:** 2022-09-21

**Authors:** Amna Devi, Romit Seth, Mamta Masand, Gopal Singh, Ashlesha Holkar, Shikha Sharma, Ashok Singh, Ram Kumar Sharma

**Affiliations:** 1 Biotechnology Department, CSIR-Institute of Himalayan Bioresource Technology (CSIR-IHBT), Palampur 176061, Himachal Pradesh, India; 2 Academy of Scientific and Innovative Research (AcSIR), Ghaziabad 201002, Uttar Pradesh, India; 3 Environmental Technology, CSIR-Institute of Himalayan Bioresource Technology (CSIR-IHBT), Palampur 176061, Himachal Pradesh, India

**Keywords:** *Angelica glauca*, transcriptome, specialized metabolites, phthalide, ferulic acid

## Abstract

*Angelica glauca* Edgew, which is an endangered medicinal and aromatic herb, is a rich source of numerous industrially important bioactive metabolites, including terpenoids, phenolics, and phthalides. Nevertheless, genomic interventions for the sustainable utilization and restoration of its genetic resources are greatly offset due to the scarcity of the genomic resources and key regulators of the underlying specialized metabolism. To unravel the global atlas of the specialized metabolism, the first spatial transcriptome sequencing of the leaf, stem, and root generated 109 million high-quality paired-end reads, assembled de novo into 81,162 unigenes, which exhibit a 61.53% significant homology with the six public protein databases. The organ-specific clustering grouped 1136 differentially expressed unigenes into four subclusters differentially enriched in the leaf, stem, and root tissues. The prediction of the transcriptional-interactome network by integrating enriched gene ontology (GO) and the KEGG metabolic pathways identified the key regulatory unigenes that correspond to terpenoid, flavonoid, and carotenoid biosynthesis in the leaf tissue, followed by the stem and root tissues. Furthermore, the stem and root-specific significant enrichments of phenylalanine ammonia lyase (PAL), cinnamate-4-hydroxylase (C4H), and caffeic acid 3-O-methyltransferase (COMT) indicate that phenylalanine mediated the ferulic acid biosynthesis in the stem and root. However, the root-specific expressions of NADPH-dependent alkenal/one oxidoreductase (NADPH-AOR), S-adenosyl-L-methionine-dependent methyltransferases (SDMs), polyketide cyclase (PKC), and CYP72A15 suggest the “root” as the primary site of phthalide biosynthesis. Additionally, the GC-MS and UPLC analyses corresponded to the organ-specific gene expressions, with higher contents of limonene and phthalide compounds in the roots, while there was a higher accumulation of ferulic acid in the stem, followed by in the root and leaf tissues. The first comprehensive genomic resource with an array of candidate genes of the key metabolic pathways can be potentially utilized for the targeted upscaling of aromatic and pharmaceutically important bioactive metabolites. This will also expedite genomic-assisted conservation and breeding strategies for the revival of the endangered *A. glauca*.

## 1. Introduction 

The Indian Himalayan Region (IHR) is an important biodiversity hotspot, with an abundance of 1748 medicinal and aromatic plant species (MAPs) that have significant utility in Indian traditional medicines [1,2]. The gradual rising demands for these MAPs have resulted in the destructive harvesting and depletion of their natural genetic resources, consequently shifting them onto the IUCN’s RET list [3,4,5,6].

*Angelica glauca* Edgew (syn: *Angelica nuristanica*; family: Apiaceae; common names: “Chora”, “Gandhrayan”, and “Smooth angelica”) is an important medicinal and aromatic herb that is endemic to the IHR. It is an endangered perennial diploid (2n = 22) herb with an extensively larger genome size (11.7 Gb), and it is distributed throughout the Western Himalayan regions at an altitudinal range of 2000–3800 m above sea level (amsl) [7,8,9,10]. The plant is glabrous, tall, and erect, having 1–3 pinnately compound leaves, with a thick fistular stem and long conic roots. The clustered flowers in the form of umbel inflorescence are among the main characteristics of the plant, which attain maturity from July to August, mediated through cross-pollination [9,11,12].

The active accumulations of phthalide (butylidene phthalide and ligustilide), ferulic acid, and terpenoids (nerolidol, menthol, and carvone) have been traditionally used for the treatment of various disorders (cardiac, renal, digestive, pulmonary, rheumatism, menorrhea, cancer, and inflammation), and they have been used widely in aromatherapy [8,12,13,14,15]. Phthalide biosynthesis involves either the condensation of malonyl CoA with acetyl-CoA or propionyl-CoA molecules, followed by the SAM-methylation-mediated production of tetraketide intermediate. Subsequently, several biochemical reactions, including cyclization, hydroxylation, carboxylation, and reduction, result in the accumulation of butylidene phthalide and ligustilide [16,17]. Similarly, the phenylpropanoid-mediated ferulic acid-biosynthesis pathway consists of the involvement of key enzymes, including phenylalanine ammonia lyase (PAL), cinnamic acid 4-hydroxylase (C4H), coumarate 3-hydroxylase (C3H), and caffeic acid 3-O-methyltransferase (COMT) [18,19]. In plants, the cytosolic MVA pathway has been suggested to contribute to sesqui- and triterpene biosynthesis, while the plastidial MEP pathway mainly synthesizes mono- and diterpenes, with limited crosstalk with the MVA pathway [20]. Nevertheless, most of these inferences were derived based on preliminary phytochemical and pharmacological studies [14,21]. Having evolved with a highly heterozygous complex genome (11.7 GB), *A. glauca* has possibly been omitted from global whole-genome-sequencing (WGS) efforts [7,10,22]. The paradigm shifts in sequencing technologies with the availability of multiple cost-effective high-throughput next-generation-sequencing (NGS) platforms has enormously contributed towards unraveling the functional and genomic insights into the specialized metabolism, irrespective of model or nonmodel plants [23,24], wherein organ-specific spatial-transcriptome-sequencing profiling has been proven to be an effective approach to the creation of comprehensive genomic resources and the elucidation of the complex biological/regulatory networks involved in the biosynthesis and transport of the specialized metabolism [25,26,27].

Therefore, global organ-specific transcriptome-sequencing efforts have been carried out for the creation of comprehensive genomic resources in the endangered Himalayan plant species *A. glauca*. Subsequently, the integration of spatial gene expressions with predicted protein interactome network analysis has assisted in the identification of the organ-specific key genes and regulators involved in the terpenoid, ferulic acid, and phthalide biosynthesis pathways with aromatic and pharmaceutical importance. The inferences derived in the current study will be a footstep for futuristic functional genomics and synthetic biology efforts for the nondestructive and sustainable production of the bioactive metabolites of *A. glauca*. In addition, novel genomic-resource creation can be utilized for the development of functionally relevant molecular-marker resources to strategize the conservation and domestication efforts in *A. glauca.*

## 2. Results

### 2.1. Transcriptome Sequencing, De Novo Assembly, and Functional Annotation

**Figure 1:**
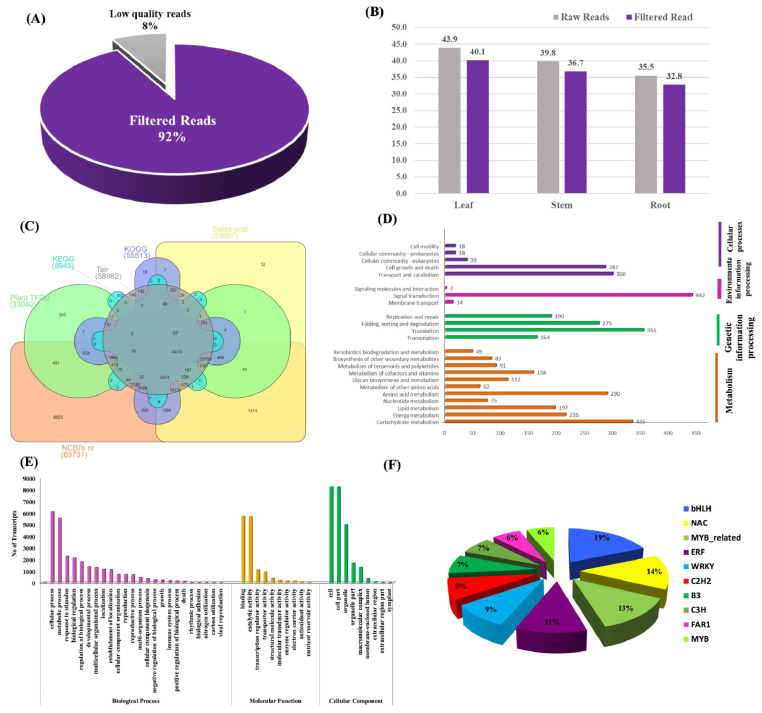
(**A**) Quality filtering of sequenced reads. (**B**) High-quality filtered reads in leaf, stem, and root tissues. (**C**) Venn diagram representing the functional annotations of transcripts with six public databases. (**D**) KEGG annotation grouped transcripts into four main categories: metabolism, genetic-information processing, environmental-information processing, and cellular processes. (**E**) Histogram representing gene ontology (GO) grouped into three functional categories: biological processes, molecular functions, and cellular components. (**F**) Pie chart representing classification of major transcription-factor families.

The organ-specific spatial transcriptome sequencing of the leaf, stem, and roots generated a total of 119 million raw reads. Quality filtering and the de novo assembly of 109 million high-quality reads resulted in 81,162 unigenes and 36,052 isoforms, with N_50_ and average transcript lengths of 1607 bp and 923.32 bp, respectively. The function assignment with six public databases annotated 72,120 (61.53%) transcripts into NCBI nr (69,731; 59.49%), TAIR (58,982; 50.31%), KOG (55,513; 47.36%), Swiss-Prot (53,957; 46.03%), Plant TFIIdb (33,040; 28.18%), and KEGG (8945; 7.63%) (Figure 1A–C, Table 1, Table 2 and Table 3 and Table S1).

The KEGG-pathway curation assigned 397 enriched metabolic pathways with top representations of metabolism (1668), genetic-information processing (984), the cellular processes (662), and environmental-information processing (458), including the transcripts that correspond to terpenoids and polyketides (91) and secondary-metabolite biosynthesis (83). Among the secondary-metabolite biosynthesis, terpenoid-backbone biosynthesis (ko00900; 30) represented the largest group, followed by phenylpropanoid biosynthesis (ko00940; 20), carotenoid biosynthesis (ko00906; 18), and flavonoid biosynthesis (ko00941; 11) (Figure 1D). 

In addition, KOG annotated 55,513 transcripts that represent 25 different major groups, exhibiting the maximum enrichment of the ‘general function prediction only’ (8117), followed by the ‘signal transduction mechanisms’ (4821), ‘transcription’ (4057), ‘posttranslational modification, protein turnover, chaperones’ (3302), and ‘secondary metabolites biosynthesis, transport and catabolism’ (1875).

Furthermore, the GO annotation classified 17,293 transcripts into 66,091 GO terms, including biological processes (26,794; 24 groups), molecular functions (14,418; 10 groups), and cellular components (24,879; 9 groups). Furthermore, the majority of the transcripts were grouped into cellular processes (6105), binding (5691), and cell including cell part (8206) in the biological processes, molecular functions, and cellular components, respectively (Figure 1E).

### 2.2. Differential Transcript Expression and Enrichment Analysis

**Figure 2:**
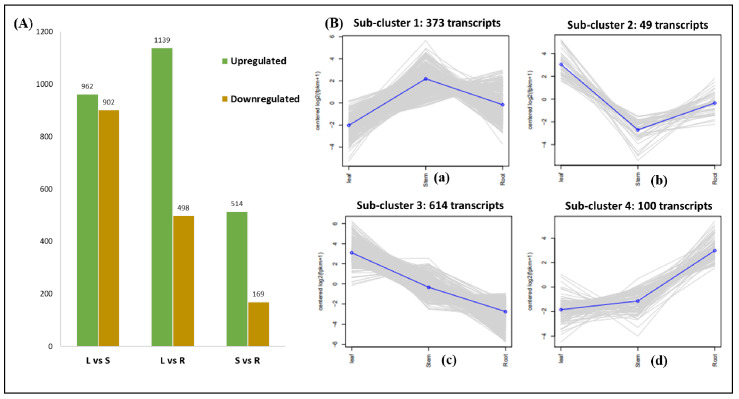
(**A**) Histogram representing significant DEGs in three combinations: leaf vs. stem (L vs. S), leaf vs. root (L vs. R), and stem vs. root (S vs. R). (**B**) Clustering of 1136 significant transcripts in leaf, stem, and root: (**a**) Subcluster 1 represents higher expressions of transcripts in the stem; (**b**) Subcluster 2 in the leaf; (**c**) Subcluster 3 in the leaf; (**d**) Subcluster 4 in the root. The color line in subfigure B represents the median values.

Pairwise combinations identified 4184 transcripts exhibiting significant differential expressions in leaf vs. stem (1894: Ups: 962; DOWNs: 902), leaf vs. root (1637: Ups: 1139; DOWNs: 498), and stem vs. root (683: Ups: 514; DOWNs: 169) (Figure 2A, Table S2). Furthermore, the clustering of 1136 key DE transcripts was categorized into four subclusters representing 373 (Subcluster 1), 49 (Subcluster 2), 614 (Subcluster 3), and 100 (Subcluster 4) transcripts. Subclusters 2 and 3 represent the transcripts significantly enriched in the leaf, while Subclusters 1 and 4 were enriched solitarily in the stem and root tissues, respectively (Figure 2B, Table S3).

#### GO-Enrichment Analysis

**Figure 3:**
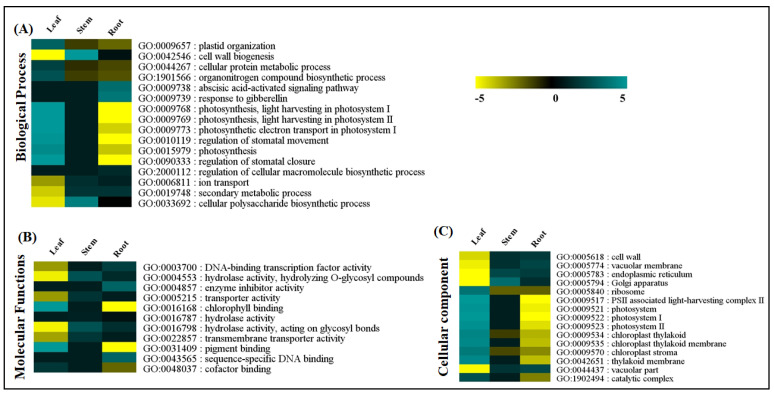
Heatmap representing GO enrichment classified into three categories: (**A**) biological processes; (**B**) molecular functions; (**C**) cellular components, in leaf, stem, and root tissues of *A. glauca*.

The organ-specific GO-enrichment analysis provided insights into the transcriptional dynamics of the significantly enriched transcripts in the leaf, stem, and root tissues of *A. glauca*. Among the biological processes, the response to gibberellin (GO:0009739), the abscisic acid-activated signaling pathway (GO:0009738), and the regulation of the cellular macromolecule biosynthetic process (GO:2000112) were recorded with significantly higher enrichments in the root tissues. However, the photosynthesis-related GO terms (GO:0009769, GO:0009768, GO:0009773, GO:0015979, GO:0009657), along with stomatal regulation (GO:0090333, GO:0010119), the cellular protein metabolic process (GO:0044267), and the organonitrogen compound biosynthetic process (GO:1901566) were significantly enriched in the leaf. Meanwhile, the stem exhibited a significantly higher enrichment of cell-wall biogenesis (GO:0042546), the cellular polysaccharide biosynthetic process (GO:0033692), and ion transport (GO:0006811). Interestingly, the secondary metabolic process (GO:0019748) showed significant enrichment in both the stem and root tissues (Figure 3A, Table S4).

Among the molecular functions, the GO terms corresponding to the transcription-factor (GO:0003700, GO:0043565) and enzyme-inhibitor (GO:0004857) activities were enriched in the root tissue. Moreover, the hydrolase (GO:0016798, GO:0004553, GO:0016787), transporter (GO:0022857, GO:0005215), and transferase (GO:0016740) activities were significantly enriched in both the stem and root tissues. The GO terms related to binding, including pigment binding (GO:0031409), chlorophyll binding (GO:0016168), and cofactor binding (GO:0048037), were enriched in the leaf tissue (Figure 3B, Table S4).

Among the cellular components, the vacuolar part (GO:0044437, GO:0005774), endoplasmic reticulum (GO:0005783), cell wall (GO:0005618), and Golgi apparatus (GO:0005794) were significantly enriched in the stem and root. In concordance with the biological processes, the leaf tissue recorded significantly higher enrichments of chloroplast thylakoid related (GO:0009535, GO:0042651, GO:0009534, GO:0009570), along with photosystems (GO:0009517, GO:0009521, GO:0009522, GO:0009523), catalytic complexes (GO:1902494), and ribosomes (GO:0005840) (Figure 3C, Table S4).

### 2.3. Protein–Protein-Interactome-Network Analysis

**Figure 4:**
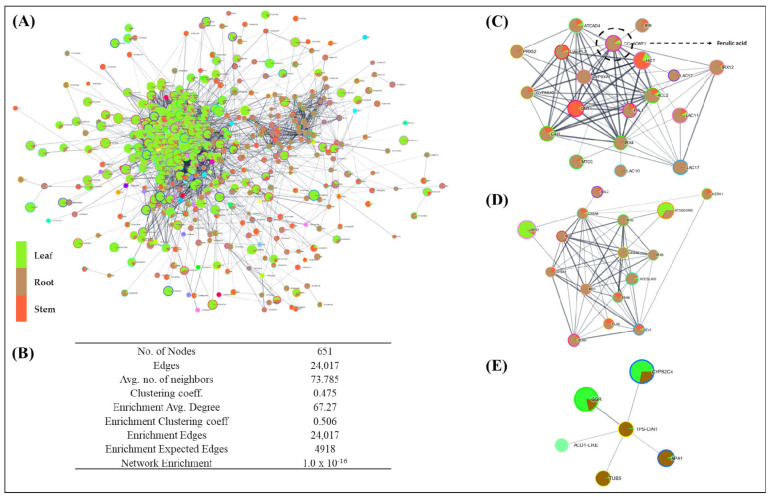
(**A**) Predicted spatial transcriptional interactome of *A. glauca*. (**B**) Network statistics of the interactome build. (**C**) Enriched network of ferulic acid pathway. (**D**,**E**) Terpenoid biosynthesis. The green color represents significant enrichment in the leaf tissue, orange in the stem tissue, and brown in the root tissue.

The protein–protein-network prediction recorded significant interactions of 651 nodes (unigenes), having 24,017 edges (average number of neighbors: 73.785; coefficient: 0.475). The predicted network revealed a significantly higher enrichment in the leaf tissue, followed by the root and stem tissues (1 × 10^−16^; average degree: 67.27; Figure 4A,B; Table S5). Interestingly, the key candidates, including caffeoyl CoA methyl transferase, having significant direct interactions with CYPs (CYP84 and CYP98), phenylalanine ammonia lyase (PAL), cinnamoyl CoA reductase (IRX4), and 4-coumarate: CoA ligase (4CL2), with significantly high enrichment in the root tissues, suggests phenylpropanoid-pathway-mediated ferulic acid biosynthesis (Figure 4C). Furthermore, the terpenoid biosynthesis recorded a significantly higher enrichment in the leaf and root, whereas terpene synthase (TPS), exhibiting a direct interaction with CYP82, Tubulin (TUB5), and Aspartic protease (APS1), exhibited higher enrichment in the roots, while the photosynthesis- and chlorophyll-biosynthesis pathways were recorded with significant enrichment in the leaf (Figure 4D,E).

### 2.4. Organ-Specific Gene-Expression Elucidation of Key Metabolite Biosynthesis

**Figure 5:**
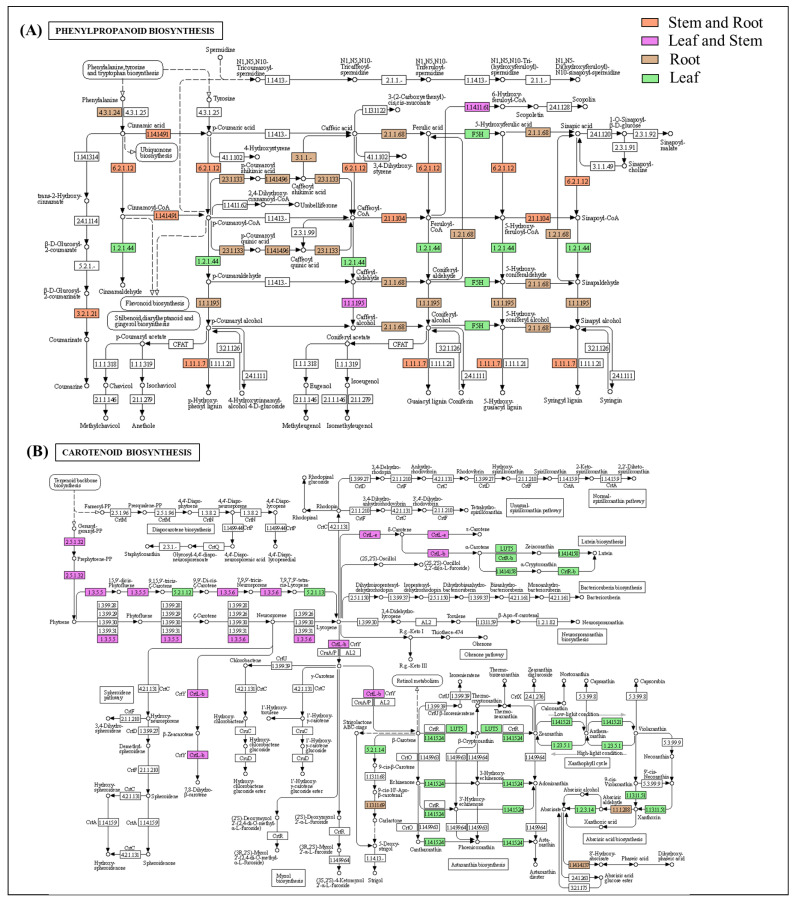
Representation of significant (**A**) phenylpropanoid- and (**B**) carotenoid-pathway enrichment in leaf, stem, and root tissues of *A. glauca*.

**Figure 6:**
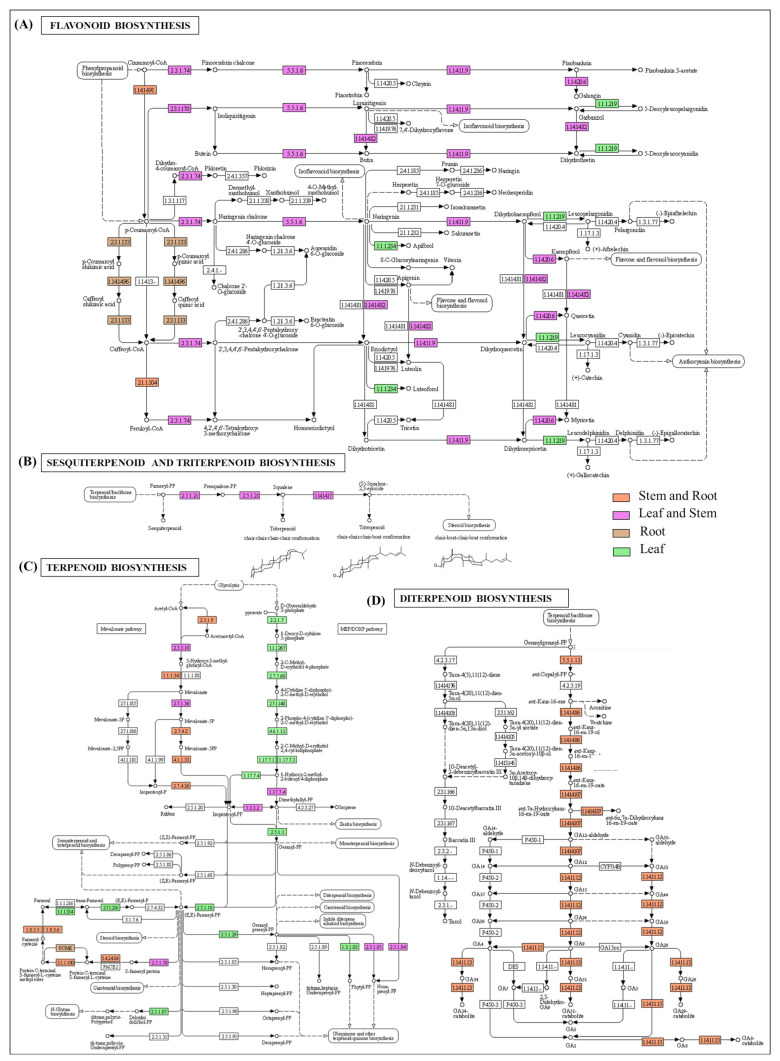
Representation of significant (**A**) flavonoids, (**B**) sesquiterpenoid and triterpenoid biosynthesis; (**C**) terpene backbone; (**D**) diterpenoids, in leaf, stem, and root tissues of *A. glauca*.

The organ-specific KEGG-pathway-enrichment analysis recorded a significantly higher enrichment of the photosynthesis-related metabolic pathways, including carbon fixation and porphyrin and chlorophyll metabolism in the leaf tissue. However, diterpenoid and phenylpropanoid biosynthesis, including ferulic acid synthesis, were found to be enriched in both the stem and root (Figure 5). Moreover, the transcripts involved in the biosynthesis of terpenoid backbone, flavonoids, and carotenoids confined their enrichment in the leaf, followed by the stem and root (Figure 5 and Figure 6). 

**Figure 7:**
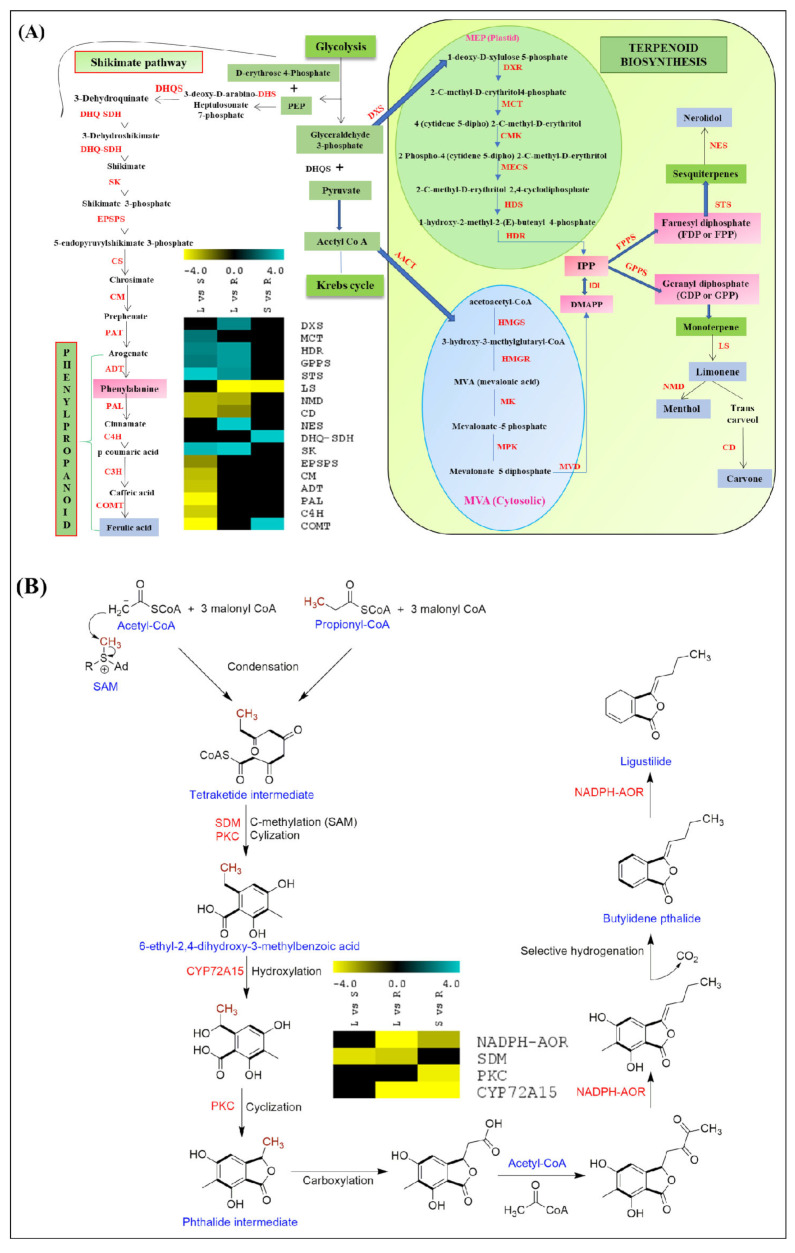
Representations of: (**A**) terpenoid and ferulic acid biosynthesis; (**B**) putative pathway of phthalide biosynthesis in *A. glauca*. Heatmap representing expression profiles of candidates across three combinations (leaf vs. stem; leaf vs. root; stem vs. root).

The root-specific higher expressions of the key candidates (NADPH-AOR, SDM, PKC, and CYP72A15) suggest the ‘root’ as the site of the synthesis and accumulation of phthalide biosynthesis. Likewise, the confined expressions of DHQ-SDH, EPSP, CM, ADT, PAL, C4H, and COMT in the stem and root complement the PPIN inference of the phenylpropanoid-pathway-mediated ferulic acid biosynthesis. Furthermore, the leaf-specific expressions of DXS, MCT, and HDR suggest the MEP-mediated biosynthesis of aromatic mono- and sesquiterpenoids. Notably, the committed step (catalyzed by GPPS) was specific to the leaf tissue, while the downstream monoterpene-diversification-related genes, including LS, NMD, and CD, exhibited higher expressions in the stem and root. Nevertheless, STS and NES, involved in sesquiterpene biosynthesis, confined their expressions in the leaf (Figure 7). This transcriptional-level study was further complemented by the metabolite profiling of the leaf, stem, and root tissues.

### 2.5. Expression Dynamics of Transcription Factors 

**Figure 8:**
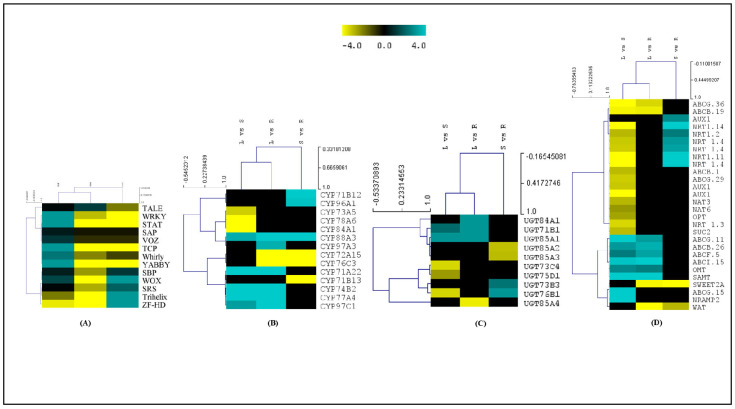
Heatmap visualization of differentially expressed transcripts of: (**A**) transcription factors (in leaf, stem, and root); (**B**) CYP; (**C**) UGT; (**D**) transporters in three combinations (L vs. S; L vs. R; S vs. R), of *A. glauca*.

Transcription factors (TFs) are key elements that regulate the spatiotemporal expression of bioactive-metabolite biosynthesis [28]. Overall, 33,040 transcripts encoding 58 TF families, with significant abundances of bHLH (3386), NAC (2439), MYB related (2398), ERF (1998), WRKY (1655), C2H2 (1456), B3 (1328), C3H (1243), FAR1 (1121), and MYB (1113), were identified in *A. glauca* (Figure 2F). Among these, 2764 TFs exhibited significant spatial expressions in the leaf (L), stem (S), and root (R), wherein bHLH (L: 65, S: 47, R: 19), NAC (L: 33, S: 54, R: 16), MYB related (L: 42, S: 46, R: 24), ERF (L: 77, S: 71, R: 22), and WRKY (L: 18, S: 20, R: 4) were the most abundant (Table 4 and Table S6). Additionally, the Trihelix, TCP, YABBY, WOX, SBP, and SRS TF families also recorded tissue-specific expressions, with higher enrichments of WRKY, TCP, and YABBY in the leaf tissue, while Trihelix, WOX, SBP, and SRS showed significant enrichments in the root tissue in *A. glauca* (Figure 8A).

### 2.6. Differential Expressions of CYPs, UGTs, and Transporters 

The CYPs and UGTs, which are a specific class of oxidoreductases and transferases, have been proven to be multifunctional candidates that regulate the biosynthesis and diversification of secondary metabolites [29,30]. In total, 602 transcripts encoding 44 CYP families were identified in *A. glauca*, with abundances of CYP71, CYP72, CYP76, CYP704, and CYP81. Of these, 30 CYPs recorded significant spatial expressions, with CYP71A22, CYP74B2, CYP77A4, CYP82C4, CYP84A1, CYP97A3, and CYP97C1 having higher expressions in the leaf. Likewise, CYP71B12, CYP73A5, CYP78A6, and CYP96A1 exhibited higher expressions in the stem, while CYP71B13, CYP72A (10, 13, 15), CYP72C, CYP76C3, CYP716A1, CYP81D8, and CYP98A3 exhibited higher expressions in the root (Figure 8B, Table S7). 

Similarly, 264 transcripts were assigned to 21 UGT families, with the majority of them belonging to three UGT families (UGT85: 65 transcripts; UGT73: 47 transcripts; UGT76: 29 transcripts). Of these, UGT71B1, UGT84A1, and UGT85A1 recorded higher expressions in the leaf, while the stem exhibited higher expressions of UGT73B3, UGT73C4, UGT75D1, and UGT76B1. Likewise, UGT85A2, UGT85A3, and UGT85A4 showed higher expressions in the root (Figure 8C, Table S7). Considering the key role of transporters in membrane transport and the accumulation of various endogenous secondary metabolites in plants [31], interestingly, 78 differentially expressed transporters identified with the ABCB.26, ABCF.5, ABCG.11, ABCG.15, dicarboxylate transporter 1 (OMT), and SAMT1 families, exhibiting higher expressions in the leaf, while the ABCB.1, ABCG.29, sucrose-transporter (SUC2), NAT, and NRT families were significantly expressed in the stem. Transcripts encoding CAT, SWEET 2A, MATE, and WAT1-related protein recorded higher expressions in the root tissues, while ABCB.19 and ABCG.36 exhibited higher expressions in the stem compared with the root (Figure 8D, Table S7).

### 2.7. RNA-Seq Validation Using qRT-PCR

**Figure 9:**
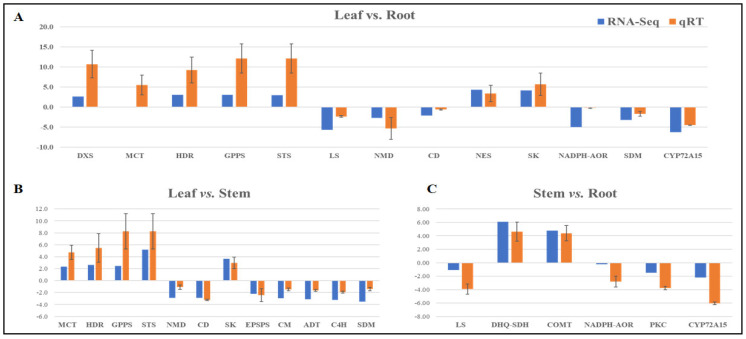
Representation of comparative expression profiles of genes (RNA-Seq and qRT) involved in specialized-metabolite-biosynthesis pathways across three combinations: (**A**) leaf vs. root; (**B**) leaf vs. stem; (**C**) stem vs. root.

The RNA-Seq expression patterns of 21 genes involved in the key metabolic biosynthesis pathways, including terpenoid (DXS, MCT, HDR, GPPS, STS, LS, NMD, CD, NES), ferulic acid (DHQ-SDH, SK, EPSPS, CM, ADT, PAL, C4H, COMT), and phthalide (NADH-AOR, SDM, PKC, CYP72A15), were well complemented by the quantitative real-time expression data, and were recorded with a positive correlation (r^2^ = 0.779) (Figure 9A–C, Table S8). However, the expression differences (0.23) between the RNA-Seq and qRT-PCR are owing to intrinsic properties, such as the detection ranges and sensitivities of these methods [32,33] (Figure 9A–C, Table S8).

**Figure 10:**
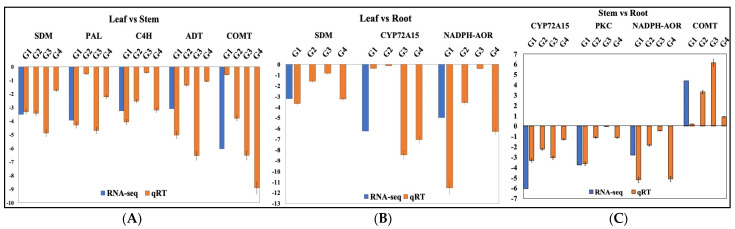
Comparative organ-specific RNA-Seq and qRT-PCR expression profiles of bioactive metabolite pathway-related genes in diverse genotypes from geographically separated populations (G1: Kinnaur_Asrang; G2: Kullu_Kandhaghai; G3: Mandi_Thunag; G4; Kullu_Gulaba) of *A. glauca* across three combintions: (**A**) leaf vs. stem; (**B**) leaf vs. root; (**C**) stem vs. root.

Furthermore, eight candidate genes (viz., ADT, PAL, C4H, COMT, NADH-AOR, SDM, PKC, and CYP72A15) were validated with four diverse genotypes of *A*. *glauca*, and the qRT-PCR expression profiles follow a similar trend of expression as that recorded in the RNA-Seq expression data (Figure 10, Table 5). However, the expression variations recorded across the genotypes may be due to the genotypic diversity and unique features of the methods.

### 2.8. GC-MS and UPLC Analysis

**Figure 11:**
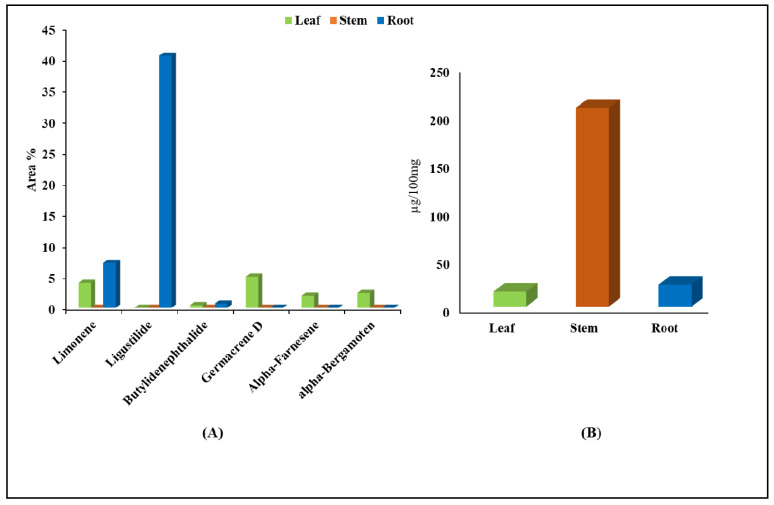
(**A**) GC-MS analysis of bioactive metabolites and (**B**) UPLC analysis of ferulic acid in leaf, stem, and root tissues of *A. glauca*.

GC-MS analysis was used to determine the volatile compounds in the leaf, stem, and root tissues of *A. glauca*. The root tissue reported higher contents of limonene, with an area percent of 7.213%, as well as ligustilide (40.48%) and butylidenephthalide (0.66%), while germacrene D (5.01%), alpha farnesene (1.96%), and alpha bergamotene, with 2.39% area, exhibited dominance presence in the leaf tissue. Moreover, the results of the UPLC analysis recorded a higher accumulation of ferulic acid in the stem (206.66 µg/100 mg), followed by the root (22.82 µg/100 mg) and leaf (15.61 µg/100 mg) tissues (Figure 11, Figures S1 and S2). 

## 3. Discussion

*A*. *glauca* is a rich repository of secondary metabolites, and especially terpenoids, phenylpropanoids, and phthalides having cardioactive, carminative, stimulant, digestive, expectorant, anti-inflammatory, and anticancerous properties are its key constituents [15,34]. Consequently, the cumulative demand of the phytopharmaceutical and aroma industries has recklessly harvested the genetic resources of *A. glauca* from its natural habitat. Additionally, soil erosion and excessive animal grazing have caused *A. glauca* to be listed among the IUCN’s endangered plants [35,36]. More recently, high-throughput cost-effective next-generation sequencing (NGS) has significantly assisted in the conservation efforts through the rapid creation of genomic resources and gene-expression patterns for the identification of the key biosynthetic pathways, irrespective of model or nonmodel medicinal plant species [23,27]. 

Therefore, in the current study, global organ-specific transcriptome sequencing was carried out, with the successful generation of 109 million paired-end reads, assembled into 81,162 unigenes in organ-specific NGS-assisted transcriptome sequencing, which were in consensus with the recent genomic efforts in related *Angelica* species [37,38]. Overall, a 61.53% functional annotation rate of the de novo assembled unigenes with public protein databases is comparable with *Ferula asafoetida* (58%) and *Thapsia laciniata* (61.9%) of the family Apiaceae [39,40]. Moreover, the integration of the predicted protein-interactome network with leaf-, stem-, and root-specific gene expressions has assisted in the identification of the key regulators for the successful elucidation of the specialized-metabolite-biosynthesis pathway.

### 3.1. Organ-Specific Transcriptional Dynamics in A. gluaca 

The global understanding of organ-specific gene expression has been successfully utilized to reveal the intricate key biosynthetic pathways and biological mechanisms, including different gene families, at the genome and transcriptional levels [41,42,43]. Significant pair-wise DEGs (leaf vs. stem; leaf vs. root; stem vs. root) and KEGG-enrichment analysis, bolstered in deciphering the complex secondary-metabolite-biosynthesis pathways in *A. glauca*, have been complemented with various previous studies, including those on *Podophyllum hexandrum* [44], *Trillium govanianum* [45], and *Fritillaria roylei* [27]. Subsequently, the prediction of the transcriptional-interactome network complementing the organ-specific expression-profiling-assisted elucidation of the complex specialized-metabolite pathways has been performed successfully in earlier studies [27,46,47,48].

The cumulative enrichment of the terpenoids, flavonoids, and carotenoids in the leaf, and the phenylpropanoid and diterpenoid biosynthesis in the root and stem tissues, suggest the intra-/intercellular trafficking of key bioactive metabolites via membrane transporters [49,50]. This was further supported by the successful prediction of the transcriptional-interactome network, which suggests phenylpropanoid-mediated ferulic acid biosynthesis in the root and stem tissues with the active involvement of caffeoyl CoA methyl transferase, phenylalanine ammonnia lyase, cinnamoyl CoA reductase, and 4-coumarate: CoA ligase. Nevertheless, the root-specific enrichment of regulators having significant interactions with the key candidates that correspond to ferulic acid synthesis further support the ‘stem and root’ as the sites of ferulic acid biosynthesis. However, the enrichment of the photosynthesis and the stomatal regulation in the predicted network in the leaf tissue indicates enough primary-metabolite flux to synthesize and accumulate secondary metabolites [51,52]. 

Furthermore, the abundance of TFs (viz., bHLH, NAC, ERF, WRKY, and MYB) indicates their role in mono- and sesquiterpenoid and phenylpropanoid biosynthesis, as reported in previous studies [53,54]. Likewise, the leaf-specific higher enrichments of WRKY, TCP, YABBY, ERF, and MYB suggest their key involvement in regulating terpenoid and flavonoid biosynthesis [28,55,56,57], while the root-specific enrichment of TFs (viz., Trihelix, WOX, and SBP) probably regulates secondary-metabolite biosynthesis [58].

### 3.2. Elucidation of Key Biosynthetic Pathways 

Phenylpropanoid biosynthesis is one of the most important pathways in secondary metabolism, and it contributes to the biosynthesis of a wide range of bioactive metabolites in plants [59]. The significant stem- and root-specific enrichments of phenylalanine ammonia lyase, cinnamate-4-hydroxylase, and caffeic acid 3-O methyl transferase suggest the tissue-specific biosynthesis and accumulation of ferulic acid. Considering that isoprenoids (C-5 isopentenyl diphosphate (IPP), and its isomer dimethylallyl diphosphate (DMAPP) act as the precursors for the terpenoid pool in plant systems, the significant KEGG enrichment of the cytosolic MVA pathway in the stem and root tissues suggests MVA-mediated diterpenoid biosynthesis [20,60]. Nevertheless, the higher enrichments of the transcripts involved in the MEP pathway in the leaf suggests MEP-mediated mono-, sesqui-, and triterpenoid and carotenoid biosynthesis in the leaf tissues, as reported earlier for *Salvia miltiorrhiza* [61] and *Cymbopogon winterianus* [62]. Furthermore, inferences in biosynthetic studies on the phthalide-like structure (marilone and mycophenolic acid) in fungi were used to elucidate the condensation-, methylation-, cyclization-, and hydroxylation-mediated putative biosynthesis pathway of phthalide in *A. glauca* [17,63,64,65]. The higher accumulation of ferulic acid in the stem, followed by the root and leaf tissues, observed in the UPLC analysis, well complemented both the RNA-Seq and qRT expression analyses. Moreover, the root-specific higher accumulation of limonene, ligustilide, and butylidene, well corroborated by the transcriptional dynamics reported in the current investigation, indicates the root as a storage site for the accumulation of these bioactive metabolites, as reported earlier [8,9].

The cytochrome P450 monooxygenases (CYPs) and UDP-glycosyltransferases (UGTs) are known to play an imperative role in the structural and chemical diversity of plant secondary metabolites (phenylpropanes, terpenoids, alkaloids, and carotenoids) by catalyzing numerous hydroxylation reactions [66,67,68,69]. The leaf-specific expressions of CYP71, 74, 77, 82, and 97, along with the UGTs (71, 84, and 85), suggest their key involvement in the modification of shikimate products, the 3-hydroxylation of carotenoid and flavonoid biosynthesis [70,71,72,73]. Nonetheless, the root-specific expressions of the CYP families (72, 76, 81, and 98) and UGT85 (A2, A3, and A4) signify their potential role in the biosynthesis, modification, and diversification of phthalide and phenylpropanoid biosynthesis in *A. gluaca* [74,75].

The inter-/intracellular transport of plant secondary metabolites is a critical process to attain metabolic homeostasis for the maintenance of the spatiotemporal biosynthesis and storage of plant secondary metabolites [47]. The leaf-specific expressions of membrane transporters, such as ABCB.26, ABCF.5, and ABCG, indicate the involvement in the cellular transport of mono-sesquiterpenoids and volatile compounds [48,76]. However, the stem-specific enrichments of the ABCB.1, ABCG.29, and NRT families, while SWEET14 and MATE were expressed in the root, indicate their role in the metabolite-flux transportation from the biosynthetic sites to the storage site [31,48].

## 4. Methods and Materials

### 4.1. Plant Materials and RNA Isolation

**Figure 12:**
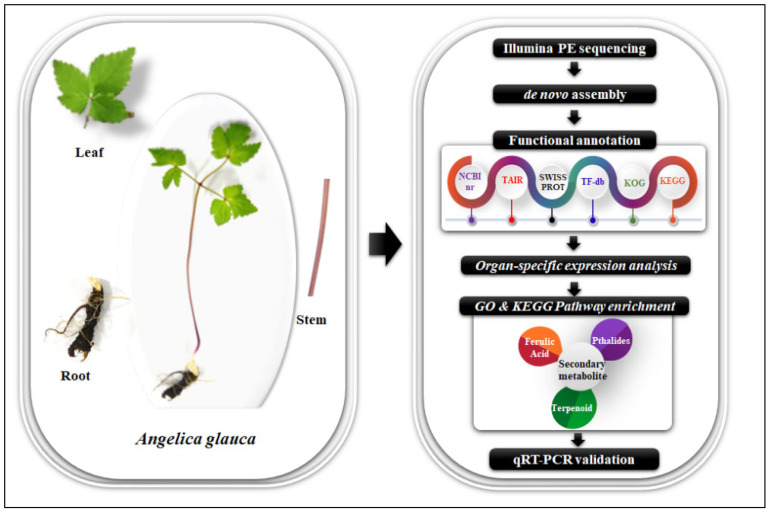
Schematic representation of methodology used for tissue-specific (leaf, stem, and root) transcriptomics study on *A. glauca*.

For the tissue-specific transcriptomic sequencing, leaf, stem, and root tissues of *A. glauca* were collected from Ranikot, Chamba district, Himachal Pradesh, India (32.5601° N, 76.1049° E), at an elevation of 2708 amsl. The plant samples were snap-frozen in liquid nitrogen and stored at −80 °C till RNA isolation. Total high-quality RNA was extracted using the iRIS protocol [77]. The concentration of RNA was determined using a NanoDrop 2000 spectrophotometer (Thermo Scientific, Waltham, MA, USA), and the quality was checked in 1% formaldehyde MOPS gel electrophoresis. The integrity of the RNA was measured in terms of the RNA-integrity-number (RIN) value using an Agilent 2100 Bioanalyzer (Agilent Technologies, Santa Clara, CA, USA), and samples having RIN values ≥ 7.0 were subsequently utilized for library preparation. The methodology adopted in this study is represented in Figure 12.

### 4.2. Library Construction, Transcriptome Sequencing, and De Novo Assembly

Three RNA-Seq libraries corresponding to the leaf, stem, and root tissues were prepared using the Illumina TruSeq RNA sample prep kit v2 (Illumina Inc., San Diego, CA, USA), following the manufacturer’s instructions, targeting 200–300 bp insert-sized libraries. The prepared libraries were quantified using a Qubit 2.0 Fluorimeter (Invitrogen, Waltham, MA, USA), followed by a quality assessment using an Agilent 2100 Bioanalyzer (Agilent Technologies, Santa Clara, CA, USA). The paired-end sequencing (2 × 100 bp) was performed using the NovaSeq 6000 platform (Illumina Inc., San Diego, CA, USA). The raw sequencing data obtained from all three tissues were deposited to the SRA (Sequence Read Archive) in the NCBI database, with the accession number SRP359017, under Bio Project PRJNA 804685. The high-quality clean reads having Phred scores (Q value) > 30 were extracted using the NGS Toolkit [78], and they were subsequently assembled (de novo) using Trinity RNA-Seq version 2.4.0 with default parameters [79]. Furthermore, the assembled sequences were clustered based on a 90% sequence homology using CD-HIT-EST version 4.6 [80]. 

### 4.3. Functional Annotation of De Novo-Assembled Transcripts

The functional annotations of the nonredundant (NR) assembled transcripts were performed with the six publicly available protein databases, including NCBI nr, Arabidopsis proteome (TAIR), Swiss-Prot, KEGG, KOG, and Plant TFII-db, using the BLASTx algorithm, with an e-value cutoff ≤ 1 × 10^−5^ [81]. AgriGO version 1.2 was used for the gene-ontology (GO) annotation, classifying transcripts into three major categories (viz., biological processes, cellular components, and molecular functions [82]). The pathway-curation and enrichment analyses were performed using the KEGG pathway-annotation database accessed on 2 July 2020 (https://www.genome.jp/kegg) [83,84]. The CYP and UGT families were identified using the *Arabidopsis thaliana* database (http://www.p450.kvl.dk/index.shtml (accessed on 2 July 2020)). 

### 4.4. Differential Expression Analysis

To determine the tissue specificity of the differential-gene-expression patterns, the high-quality reads were mapped onto the NR transcripts using the Bowtie 2 tool [85]. The reads mapped onto the assembled transcripts were quantified in terms of FPKM using the RSEM tool [86]. Furthermore, the EdgeR package was used to assess the differential transcript expressions in the following pair-wise comparisons: leaf vs. stem; leaf vs. root; stem vs. root, with the false-discovery rate (FDR < 0.05) and log_2_ fold change (≥2) as the differentially expressed genes [87]. The differential-pathway-enrichment analysis was performed by gene-enrichment analysis using the R Bioconductor package, as previously used [46]. The Fischer exact test (Hochberg FDR adjustment cutoff < 0.05) was used for the identification of the organ-specific significant differentially enriched pathways. 

### 4.5. Protein–Protein-Interactome-Network Prediction

The protein–protein-interactome network was predicted by using the predetermined PPI network of *Arabidopsis thaliana* [88]. The orthologs of the significant organ-specific enriched transcripts were obtained with the TAIR database using the BLASTx tool. The interactome network was built and analyzed using Cytoscape ver. 3.4.0 an open-source software maintained by National Institute of General Medical Sciences (NIGMS), U.S [89]. The nodes of the network represent transcripts, with the respective orthologs having significant correlation edges (FDR ≤ 0.05) of the predicted network. The R Bioconductor package was used to perform the pathway curation and gene-set enrichment of the predicted network [90].

### 4.6. qRT-PCR Validation of RNA-Seq Data

The validation of the RNA-Seq data was performed with the key genes of the pathways using qRT-PCR analysis (ST 8). Gene-specific primers were designed using Batch Primer 3 ver1.0 (Albany, NY, US) (http://probes.pw.usda.gov/batchprimer3/ (accessed on 2 July 2020)).

The cDNA was prepared from 2 μg of high-quality total RNA using a cDNA synthesis kit (Thermo Scientific, Waltham, MA, USA, Revert H Minus). The reaction mixture was composed of 1μL of template cDNA, 0.5 μL of gene-specific primers, 5 μL Power SYBR Green mix (Applied Biosystems, Thermo Fisher Scientific, Waltham, MA, USA), and 3 μL nuclease-free water, and the amplifications were performed in the real-time PCR system (QuantStudio 5, Applied Biosystems, Waltham, MA, USA). The relative expression levels of the genes were calculated from the cycle-threshold (ct) values using the 2^−ΔΔct^ method, considering EF-1α as a reference gene [91]. Additionally, the expression stabilities of the key candidate genes were validated in four diverse genotypes of *A. glauca*, collected from the natural habitats of the geographically separated populations of Western Himalaya, India (viz., Kinnaur_Asrang (G1: 3376 m, 31.667° N, 78.313° E); Kullu_Kandhaghai (G2: 2250 m, 31.408° N, 77.444° E); Mandi_Thunag (G3: 2177 m, 31.577° N, 77.152° E); Kullu_Gulaba (G4: 2521 m, 32.317° N, 77.189° E) (Table 5)).

### 4.7. Metabolite Profiling

The phytochemical analysis of the specialized metabolites in the leaf, stem, and root tissues was performed using gas chromatography–mass spectrometry (GC-MS) and ultra-performance liquid chromatography (UPLC-MS). Volatile compounds were extracted and analyzed by a mass spectrometer (QP2010 Shimadzu, Tokyo, Japan), attached with an AOC-5000 auto injector and a DB-5 (SGE International, Ringwood, Australia) fused-silica capillary column of a 30 m length, 0.25 mm i.d., and 0.25 μm film thickness. Helium was utilized as a carrier gas, with a constant flow rate of 1.1 mL/min, and the program was set as in a prior study [92]. 

Ultra-performance liquid chromatography (UPLC) was used to identify the ferulic acid, and the analysis was carried out using the Water Acquity UPLC-H class system, which includes a binary solvent manager, an autosampler, a column heater, and an eλ photodiode array detector (PDA). All the parameters used in the analysis followed a previous study [93], and the chromatographic data were recorded for each sample and were quantified based on internal standard curves.

## 5. Conclusions

The specialized metabolites of the high-altitude Himalayan endangered species *A*. *glauca* are a vital resource for the pharmaceutical and aromatic industries. The increasing demand has led to the indeterminate extraction of natural plant populations. Therefore, the creation of the first NGS-assisted comprehensive spatial genomic resource has assisted in the identification of the tissue-specific key regulators that influence the specialized-metabolite biosynthetic pathways in *A*. *glauca*, which will enable the identification of the targeted secondary-metabolite-biosynthesis pathways in other medicinal plants. Furthermore, the tissue-specific molecular insights into the terpenoid, ferulic acid, and phthalide biosynthesis will assist leaf-, stem-, and root-specific genetic interventions to upscale the targeted metabolite synthesis with the active involvement of TFs, CYPs, UGTs, and transporters that correspond to the biosynthesis, transportation, and diversification of secondary metabolites. The present study paves the way to understand the multifarious metabolic genes for the synthesis of diverse biomolecules in plant species. Creating genomic resources from the current study will formulate future steps to enhance metabolite production by genetic engineering and molecular functionally relevant marker resources for the population diversity and conservation planning of this important Himalayan medicinal plant. 

## Figures and Tables

**Table 1:** Total raw and filtered reads generated for leaf, stem, and root tissues of *A. glauca*.

S. No.	Sample Name	Raw Reads	Filtered Reads	Retained High-Quality Reads (%)
1	Leaf	43,893,016	40,144,376	91.4
2	Stem	39,814,592	36,737,576	92.2
3	Root	35,495,806	32,757,628	92.2
	Total reads	119,203,414	109,639,580	91.9

**Table 2:** De novo-assembly statistics of *A. glauca* transcriptome.

De Novo-Assembly Statistics		
	Before Clustering	After Clustering
Total transcripts	1,61,836	1,17,214
Total genes	81,570	81,162
Percent GC	39.69	39.62
Contig N50	1751	1607 bp
Median contig length	715	526 bp
Average contig length	1071.45	923.32 bp

**Table 3:** Functional annotations of transcripts with various public databases.

Database	No. of Transcripts	Percent (%)
NCBI nr	69,731	59.49
TAIR	58,982	50.31
KOG	55,513	47.36
Swiss-Prot	53,957	46.03
Plant TFDB	33,040	28.18
KEGG	8945	7.63
Total	72,120	61.53

**Table 4:** Tissue-specific expressions of abundant TFs encoding transcripts in *A. glauca*.

S. No.	TF Family	Leaf (L)	Stem (S)	Root (R)
1	bHLH	65	47	19
2	NAC	33	54	16
3	MYB related	42	46	24
4	ERF	77	71	22
5	WRKY	18	20	4
6	C2H2	20	21	9
7	B3	33	14	9
8	C3H	16	23	2
9	FAR1	21	16	14
10	MYB	17	21	5

**Table 5:** Geographical description of four diverse genotypes of *A. glauca* from Himachal Pradesh.

S. No.	District	Population	Latitude (°N)	Longitude (°E)	Altitude (m)
1	Kinnaur	Asrang (G1)	31.667	78.313	3376
2	Kullu	Kandhaghai (G2)	31.408	77.444	2250
3	Mandi	Thunag (G3)	31.577	77.152	2177
4	Kullu	Gulaba (G4)	32.317	77.189	2521

## Data Availability

The raw reads were deposited to the NCBI SRA database with the accession number SRP359017 under Bio Project PRJNA 804685.

## Supplementary Materials

The following supporting information can be downloaded at: https://www.mdpi.com/article/10.3390/ijms231911064/s1.

## Abbreviations

IHR	Indian Himalayan Region
MAPs	Medicinal and Aromatic Plant species
UGT	UDP-Dependent Glucuronosyltransferase
ABC	ATP-binding cassette
CYP450	Cytochrome P450
DXS	1-deoxy-D-xylulose-5-phosphate synthase
DXR	1-deoxy-D-xylulose 5-phosphate reductoisomerase
MCT	2-C-methyl-D-erythritol 4-phosphate cytidylyltransferase
CMK	4-diphosphocytidyl-2-C-methyl-D-erythritol kinase
MECS	2-C-methyl-D-erythritol 2,4-cyclodiphosphatesynthase
HDS	4-hydroxy-3-methylbut-2-enyl diphosphate synthase
HDR	4-hydroxy-3-methylbut-2-enyl diphosphate reductase
AACT	Acetyl Co-A acetyltransferase
HMGS	HMG-CoA synthase
HMGR	HMG-CoA reductase
MK	Mevalonate kinase
MPK	Phosphomevalonate kinase
MVD	Di phosphomevalonate decarboxylase
IDI	Isopentenyl-diphosphate delta-isomerase
GPPS	Geranyl pyrophosphate synthase
FPPS	Fernyl pyrophosphate synthase
STS	Sesquiterpene synthase
LS	Limonene synthase
NMD	Neomenthol dehydrogenase
CD	Carveol dehydrogenase
NES	Nerolidol synthase
DHS	3-deoxy-D-arabino-hept-2-ulosonate 7-phosphate synthase
DHQS	3-dehydroquinate synthase
DHQ-SDH	3-dehydroquinate dehydratase/shikimate 5-dehydrogenase
SK	Shikimate kinase
EPSPS	5-enolpyruvylshikimate-3-phosphate synthase
CS	Chorismate synthase
CM	Chorismate mutase
PAT	Prephenate aminotransferase
ADT	Arogenate dehydratase
PAL	Phenylalanine ammonia lyase
C4H	Cinnamate-4-hydroxylase
C3H	Coumarate 3-hydroxylase
COMT	Caffeic acid 3-O-methyltransferase
NADPH-AOR	NADPH-dependent alkenal/one oxidoreductase
SDM	S-adenosyl-L-methionine-dependent Methyltransferases
PKC	Polyketide cyclase
