# Supplementary material for: Spatial Genomic Resource Reveals Molecular Insights into Key Bioactive-Metabolite Biosynthesis in Endangered Angelica glauca Edgew
Source: Int J Mol Sci. 2022 Sep 21;23(19):11064. doi: 10.3390/ijms231911064 (PMC9569870; doi:10.3390/ijms231911064)
Supplement: Supplementary file 1 [file ijms-23-11064-s001.zip › Supplementary Figures.pdf]

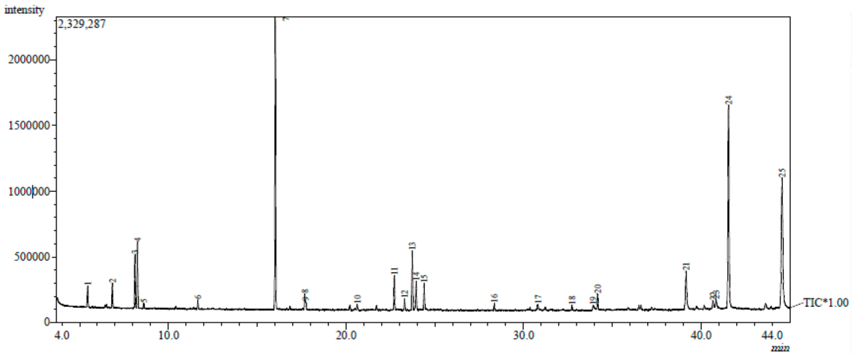

Compound	Peak	R.Time	Area	Height	Area %
Limonene	3	8.114	1263945	413714	4.01
Germacrene -D	13	23.736	1578571	452401	5.01
alpha-Bergamoten	14	23.957	753617	219773	2.39
Alpha-Farnesene	15	24.404	617615	198246	1.96
Butylidenephthalide	17	30.807	132710	38162	0.42

(A) Leaf

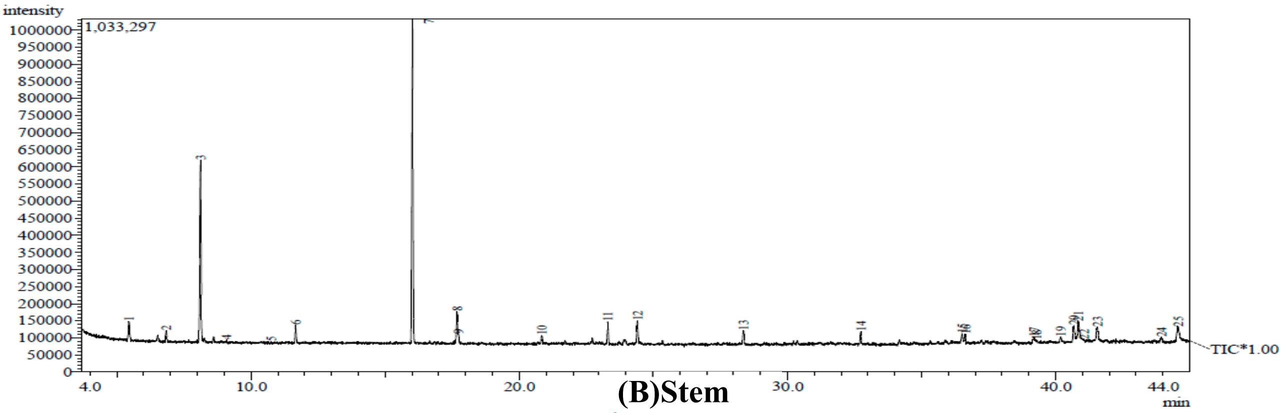

(B)Stem

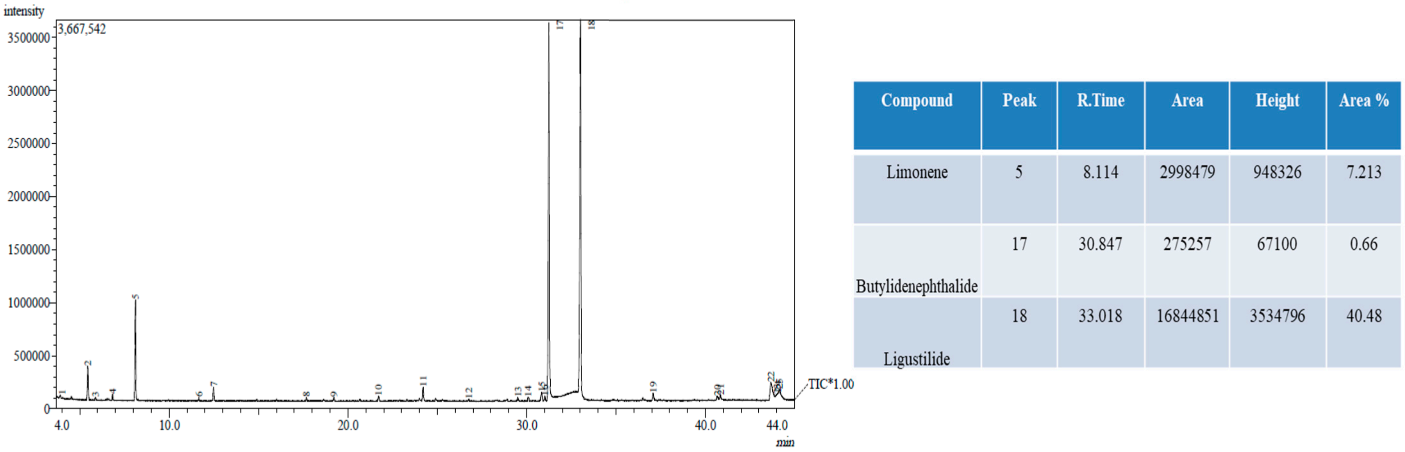

Compound	Peak	R.Time	Area	Height	Area %
Limonene	5	8.114	2998479	948326	7.213
Butylidenephthalide	17	30.847	275257	67100	0.66
Ligustilide	18	33.018	16844851	3534796	40.48

(C) Root

Figure S1. Chromatogram representing GC-MS analysis of metabolites in (A) Leaf (B) Stem and (C) Root tissues of *A. glauca*

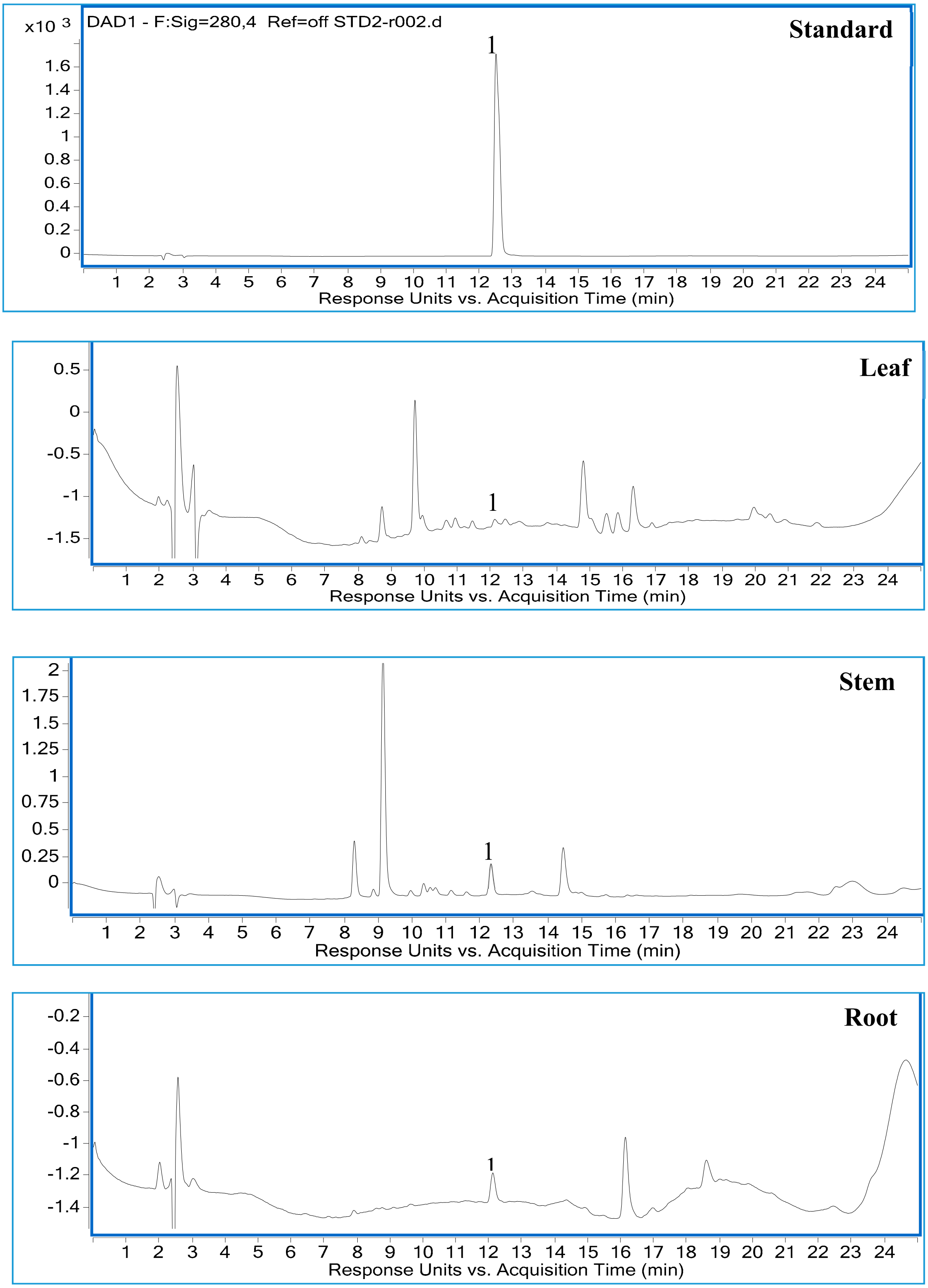

Figure S2. Chromatogram representing UPLC analysis of ferulic acid in Leaf, Stem and Root tissues of *A. glauca.*
